# Bovine Milk Triacylglycerol Regioisomer Ratio Shows Remarkable Inter-Breed and Inter-Cow Variation

**DOI:** 10.3390/molecules26133938

**Published:** 2021-06-28

**Authors:** Zhiqian Liu, Simone Rochfort

**Affiliations:** 1 Agriculture Victoria Research, AgriBio, 5 Ring Road, Bundoora, VIC 3083, Australia; Simone.Rochfort@agriculture.vic.gov.au; 2 School of Applied Systems Biology, La Trobe University, Bundoora, VIC 3083, Australia

**Keywords:** milk, triacylglycerols, regioisomers, liquid chromatography-mass spectrometry

## Abstract

Regioisomers (or positional isomers) of triacylglycerols (TAGs) of milk are known to show differential outcome in relation to human absorption. Quantitation of TAG regioisomers remains a big challenge due to the lack of facile chromatographic separation technique. The feasibility of using fragment ion intensity ratio to determine the ratio of co-eluting AAB/ABA-type regioisomer pairs was confirmed in this study. The ability of C30 stationary phase in resolving interfering TAG isomers was demonstrated for the first time. This allowed us to reveal the complexity of using fragment ion intensity to quantify 1,2-olein-3-palmitin (OOP), 1,3-olein-2-palmitin (OPO), 1,2-olein-3-stearin (OOS), and 1,3-olein-2-stearin (OSO) regioisomers in milk samples. A novel algorithm was proposed to consider the contribution of OPO/OOP and OSO/OOS double bond (DB)-isomers and to eliminate the interference of isobaric ions from other isomers, an aspect overlooked in previous studies. This liquid chromatography-mass spectrometry method that requires no pre-fractioning and a moderate chromatographic separation time of 36 min is simple and, thus, suitable for screening a large number of samples for genetic analysis of this trait. Preliminary results using a small cohort of animals showed that OPO/OOP ratio differs significantly between Jersey and Holstein cows, and a large variation was also observed across individual Holstein cows.

## 1. Introduction

Lipids are one of the major nutritive components of human and bovine milk alongside lactose and proteins, whereas triacylglycerols (TAGs) are the dominant fraction (>95%) of milk lipids [1]. TAGs are small molecules (MW < 1200 Da) containing three fatty acids (FAs) esterified to the three positions (named sn-1, sn-2 and sn-3) of glycerol backbone. At least 400 FAs are thought to be present in bovine milk [1], so hundreds of thousands of TAG species may be created by combining these FAs. Recently, over 3000 TAG species were identified in bovine milk [2]. The concentration of TAG species varies considerably, with those containing C14:0, C16:0, C18:0, C18:1 and C18:2 being the most abundant ones in both bovine and human milk [2,3].

It is widely accepted that the distribution of the three FAs at the three positions of glycerol backbone is not random in the case of milk TAGs [4,5]. For example, short-chain FAs, such as C4:0 and C6:0, are mostly found at sn-3, whereas C18:1 tends to be esterified to the sn-1 position [1]. Consequently, the existence of regioisomers (isomers differing in FA positions only, also called positional isomers) is not expected to be widespread. Indeed, whether and how many regioisomers are present remains obscure for most TAG species.

The most studied TAG species of milk is probably TAG 18:1-18:1-16:0 (a TAG molecule containing two oleic acid and one palmitic acid moieties), which is one of the most abundant species in both human and bovine milk. This TAG species is known to occur as two regioisomers in milk, namely TAG 18:1/16:0/18:1 (OPO) and TAG 18:1/18:1/16:0 (OOP) (“O” stands for oleic acid and “P” stands for palmitic acid). It has been demonstrated that the OPO isomer is favorable for infant absorption [6,7], whereas the OOP isomer, upon pancreatic lipase hydrolysis, releases free palmitic acid, which forms insoluble complexes with calcium (called calcium soap), and is no longer available for absorption [8].

The ratio of OPO vs. OOP is not equal in biological materials. While OPO is the dominant isomer in human milk (75–93%), the proportion of OPO is much lower (<60%) in bovine milk [9,10,11,12]. Being the most important commercial milk worldwide, bovine milk is used as a popular food ingredient, a common infant formula ingredient, as well as directly consumed as staple drink. So, increasing the OPO/OOP ratio of bovine milk should improve the uptake of milk lipids by a large number of people worldwide. The enhanced nutritive value that OPO isomer offers for infants may be equally applicable to humans of all ages. 

Little is known about the factors that influence the percentage of OPO regioisomer in milk. The contrasting ratio of OPO vs. OOP between human and bovine milk appears to suggest that this trait may be genetically regulated, given that oleic acid and palmitic acid are the two most abundant FAs for both human and bovine milk [13,14,15]. In order to study the genetic determinism of OPO/OOP ratio and explore the potential of using genomic selection to produce OPO-enriched bovine milk, a simple and reliable phenotyping method for screening a large number of milk samples is needed.

As TAG regioisomers have the same molecular mass and fragment ions, mass spectrometry is not able to differentiate them. As a result, chromatographic separation is essential for quantification of these isomers. Various techniques have been tested so far for regioisomer separation. For example, Kalo et al. [16] reported that normal-phase HPLC was able to separate AAB/ABA regioisomers (i.e., TAGs containing two types of FAs) with one or two short acyl (C4 or C6) chain, whereas most reversed-phase columns can only resolve regioisomers containing one double bond (DB), such as POP and PPO pair, but are unable to separate OPO/OOP pair, which contain two DBs [17]. Until now, only Ag^+^-HPLC-ELSD, in combination with a reversed-phase LC pre-fractioning in most cases, have been used successfully for the separation and quantitation of OPO/OOP in human milk and infant formula samples [10,12,18]. A simpler and a higher throughput method is still lacking.

It was found that in the case of ABA/AAB regioisomer pairs, the intensity ratio of the fragment ions (after neutral loss of one FA chain) or diacylglycerol^+^ (DAG^+^) ions (AB^+^ vs. AA^+^) is constant for each isomer but differs sharply between the two isomers [19,20]. This attribute has been explored in several reports to quantify TAG regioisomers in vegetable oils, fish oils, and animal fats without chromatographic separation [21,22,23,24,25]. The objective of this study was to evaluate the utility of this approach for the determination of OPO/OOP in milk samples and to refine the algorithm to take into consideration of the complex isomer profile of milk TAGs. The optimized method was then used to investigate the inter-breed and inter-cow variation of OPO % in bovine milk. In addition to OPO/OOP pair, another regioisomer pair OSO/OOS (“S” stands for stearic acid), which has been largely ignored so far but is expected to display a similar behavior vis-à-vis human absorption, was also included in this study. 

## 2. Results and Discussion

### 2.1. Intensity and Pattern of Fragment Ions

**Figure 1:**
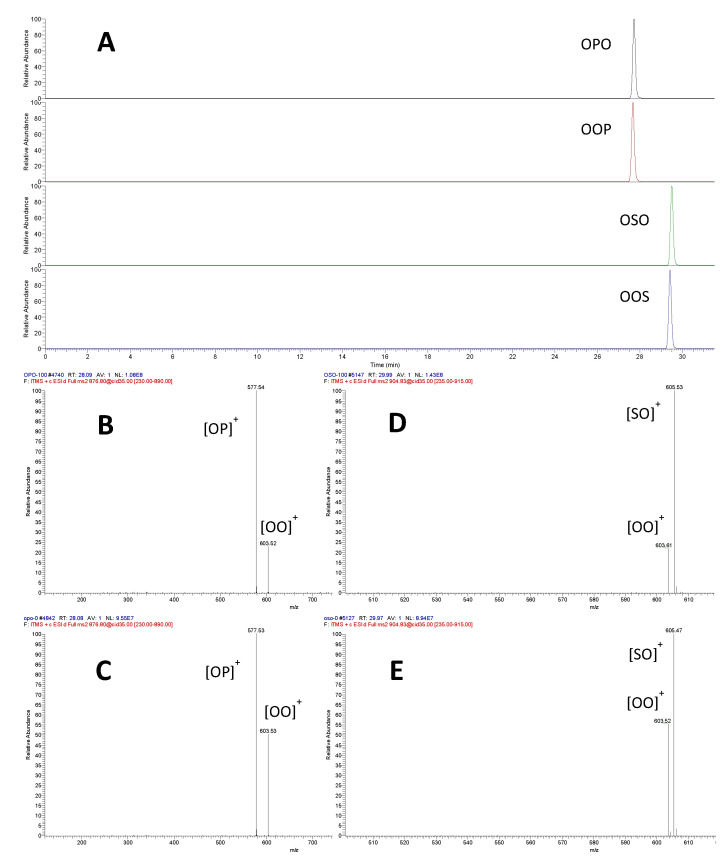
Extracted ion chromatogram (EIC) profile of OPO, OOP, OSO, and OOS standards (**A**) and their respective MS2 spectrum ((**B**) OPO; (**C**) OOP; (**D**) OSO; (**E**) OOS). P: palmitic acid; O: oleic acid; S: stearic acid. [OP]^+^, [OO]^+^, and [SO]^+^ are fragment ions or DAG^+^ ions.

Chromatographic separation of OPO/OOP and OSO/OOS pairs was not achieved with the C30 column (Figure 1A); increasing the gradient elution to 50 min did not make noticeable improvement on the separation (results not shown). Indeed, among all the reversed-phase columns used in lipidomic analysis, only a non-endcapped polymeric ODS column (Inertsil ODS-P) was reported to separate OPO/OOP pair, but a lengthy (200 min) elution was needed [9]. 

As found in previous reports [20,21], the ratio of fragment ions [OO]^+^ vs. [OP]^+^ (*m*/*z* 603.5 and 577.5, respectively) was different for the regioisomer pair, around 20% for OPO and 50% for OOP isomers (Figure 1B,C). A similar fragment ion pattern was observed with OSO/OOS pair, with a [OO]^+^ vs. [SO]^+^ ratio (*m*/*z* 603.5 and 605.5, respectively) being around 20% for OSO and 60% for OOS (Figure 1D,E).

**Figure 2:**
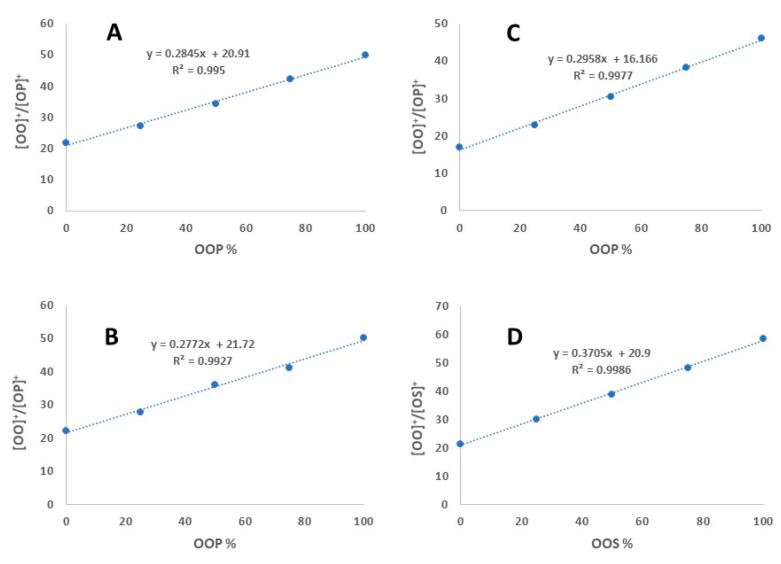
Calibration curves (fragment ion ratio vs. regioisomer %) for OPO/OOP and OSO/OOS pairs. (**A**) linear regression with 0.01 mg/mL standards (OPO/OOP) and 35 eV collision energy; (**B**) linear regression with 0.001 mg/mL standards (OPO/OOP) and 35 eV collision energy; (**C**) linear regression with 0.01 mg/mL standards (OPO/OOP) and 25 eV collision energy; (**D**) linear regression with 0.01 mg/mL standards (OSO/OOS) and 35 eV.

A series of binary mixtures of varying OPO (0.01 mg/mL) and OOP (0.01 mg/mL) proportions (100:0, 75:25, 50:50, 25:75, and 0:100) were prepared and their [OO]^+^/[OP]^+^ determined. When we plot the [OO]^+^/[OP]^+^ ratio against the % of OOP, a linear relationship (R^2^ > 0.99) was observed (Figure 2A). This linear response was maintained when a lower concentration of OPO and OOP (0.001 mg/mL each) was used (Figure 2B), or when a reduced collision energy (25 eV) was applied, although the y intercept dropped from 21 to 16 in the latter case (Figure 2C). In the case of OSO/OOS pair, a linear response was also observed between the fragment ion ratio ([OO]^+^/[SO]^+^) and the % of OOS in the mixture, but the slope is different compared to the OPO/OOP pair (Figure 2D). These results suggest that the ratio of co-eluting ABA/AAB-type regioisomers can be determined using the ratios of fragment ions. However, as the slope varies with isomer pairs, a standard calibration curve (using regiopure standards) is needed for quantification of each regioisomer pair.

### 2.2. Complexity of Milk TAG Isomer Profile

Before applying this method to milk samples, it is necessary to check the entire isomer species composition of the two TAG groups (a TAG group is defined as a series of species having the same total acyl carbon number and the same number of total double bonds), namely TAG 52:2 and TAG 54:2, that contain OPO/OOP and OSO/OOS, respectively.

**Figure 3:**
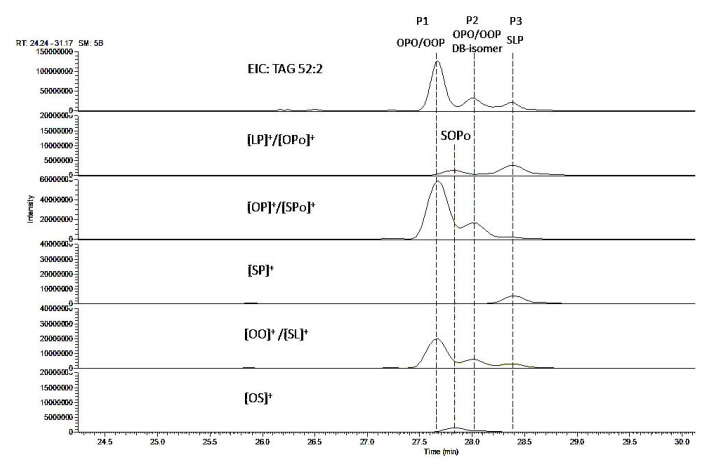
LC-MS (EIC) profile of TAG 52:2. Alignment of the parent ion (*m*/*z* 876.8) with the fragment ions *m*/*z* 575.5 ([LP]^+^ or [OPo]^+^), 577.5 ([OP]^+^ or [SPo]^+^), 579.5 ([SP]^+^), 603.5 ([OO]^+^ or [SL]^+^), and 605.5 ([OS]^+^), allowing assignment of the three peaks as OPO/OOP mixture (Peak 1), OPO/OOP DB-isomers (Peak 2), and SLP (Peak 3); noting the presence of isobaric fragment ions [LP]^+^/[OPo]^+^, [OP]^+^/[SPo]^+^, and [OO]^+^/[SL]^+^ for this TAG group. L: linoleic acid; P: palmitic acid; Po: palmitoleic acid; O: oleic acid; S: stearic acid.

The total ion chromatogram (TIC) of bovine and human milk lipids is presented in Figure S1 (Supplementary Materials). The EIC of TAG 52:2 of bovine milk shows three distinct peaks after separation with the C30 stationary phase (Figure 3). Peak 1 was identified as OPO/OOP mixture using fragment ion ([OO]^+^ and [OP]^+^) and retention time information. The same fragment ions [OO]^+^ and [OP]^+^ were detected with Peak 2, implying this peak is another isomer of OPO/OOP pair. Since only two possible regioisomers can occur with two FAs and RP-LC is not known to separate enantiomers, Peak 2 was, thus, assigned as OPO/OOP DB-isomers (DB-isomers are defined as TAGs containing C18:1 with a DB location other than Δ9). Peak 3 aligned with three fragment ions [LP]^+^, [SP]^+^, and [SL]^+^ (“L” stands for linoleic acid), corresponding to a different isomer species SLP (i.e., an isomer containing stearic acid, linoleic acid and palmitic acid). In addition, a minor isomer which was overshadowed by Peak 1 and 2 was also detected and identified as SOPo (“Po” stands for palmitoleic acid), or TAG 18:0-18:1-16:1) through alignment of fragment ions (deconvolution) (see the 2nd vertical dotted line).

Chromatographic separation and identification of extra isomer species alongside OPO/OOP pair has important implication in implementing the strategy of using fragment ion ratio (i.e., [OO]^+^ vs. [OP]^+^) to determine the ratio of OPO/OOP regioisomers. Indeed, the isomer species SLP produces fragment ion [SL]^+^, an isobaric ion of [OO]^+^, which would overestimate the intensity of [OO]^+^ and alter the [OO]^+^/[OP]^+^ ratio, if not separated with OPO/OOP. On the other hand, Peak 2 should not cause any distortion of the intensity ratio of [OO]^+^ vs. [OP]^+^ if co-eluted with Peak 1, given that the OPO/OOP DB-isomers are expected to display the same fragmentation pattern as their respective 18:1 *cis* 9 analogues. The presence of the minor isomer SOPo which co-elutes with Peak 1 and 2 further demonstrates the complexity of the isomer profile of milk TAGs, but the isobaric fragment ion [SPo]^+^ generated by this minor isomer is not expected to change significantly the intensity of [OP]^+^.

Taking into consideration of the abundance, as well as the FA composition of all the isomer species of TAG 52:2, we propose the following equation for the calculation of OPO % in milk samples:

Global OPO (%) = [(P1 area × OPO % of P1) + (P2 area × OPO % of P2)]/(total area of P1 and P2) × 100, where OPO % of P1 and P2 can be determined using the same regression equation shown in Figure 2A.

Clearly, the accuracy of the global OPO % determined using this formula depends on the peak area integration accuracy of Peak 1 and Peak 2. So, an adequate chromatographic separation of the three peaks while avoiding column overloading is of critical importance. It is worth mentioning that the resolution of the three peaks shown in Figure 3 can be further improved by increasing the gradient elution time from 25 to 50 min (Figure S2).

**Figure 4:**
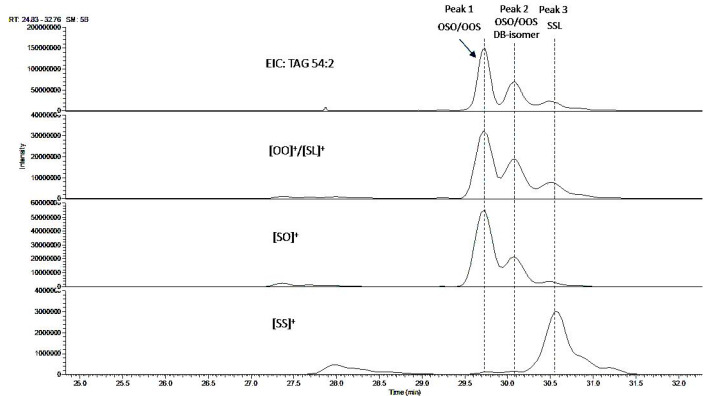
LC-MS (EIC) profile of TAG 54:2. Alignment of the parent ion (*m*/*z* 904.8) with the fragment ions *m*/*z* 603.5 ([OO]^+^ or [SL]^+^), 605.5 ([SO]^+^), and 607.5 ([SS]^+^) allowing assignment of the three peaks as OSO/OOS mixture (Peak 1), OSO/OOS DB-isomers (Peak 2), and SSL (Peak 3); noting the presence of isobaric fragment ions [OO]^+^ and [SL]^+^ for this TAG group. L: linoleic acid; O: oleic acid; S: stearic acid.

The EIC of TAG 54:2 of bovine milk also shows 3 major peaks (Figure 4). Peak 1 was identified as a mixture of OSO and OOS based on fragment ion and retention time data, while Peak 2 was assigned as DB-isomers of OSO/OOS, Peak 3 contains a different isomer species SSL (i.e., TAG 18:0-18:0-18:2). In addition to these three main isomer clusters, several minor isomers were detected through fragment ion alignment, but the contribution of their isobaric ions to the [OO]^+^/[SO]^+^ ratio is minimal (results not shown). Consequently, the same equation can be used to estimate the global OSO % in milk samples.

It is to be noted that the isomer composition and EIC profile of TAG 52:2 and TAG 54:2 of human milk is very similar to those of bovine milk. Therefore, the current method is equally applicable to the determination of OPO and OSO % of human milk samples.

Although the concept of using fragment ion intensity ratios to quantify TAG regioisomers has been explored in several studies, the potential influence of other isomers and isobaric ions was mostly neglected [26]. This may or may not be a concern depending on the matrices analyzed, as different matrices may show a very different isomer profile. In the case of milk samples, satisfactory chromatographic separation is needed to circumvent this issue, which has proven difficult to achieve with C18 stationary phases [14]. Using a C30 column in combination with non-aqueous reversed-phase elution regime, we have shown, for the first time, the complex profiles of TAG 52:2 and TAG 54:2 and the implication of isomer species in determining the % of OPO and OSO in milk samples. The separation of OOP/POO and OOS/SOO enantiomers was not attempted in this work, which appears to be possible only with chiral columns [27,28].

### 2.3. Repeatability

When our method was applied to human and bovine milk samples, satisfactory measurement repeatability (as judged by the RSD of repeated analyses) was observed for OPO % (RSD < 2%), as well as OSO % (RSD < 3%), with both human and bovine milk samples (Table 1). It is worth noting that very similar values were obtained with both neat and 10-fold diluted milk samples. In addition, our results on OPO % in human and bovine milk are in good agreement with those reported previously [9,10].

### 2.4. Inter-Breed Variation of Regioisomer Ratios

**Figure 5:**
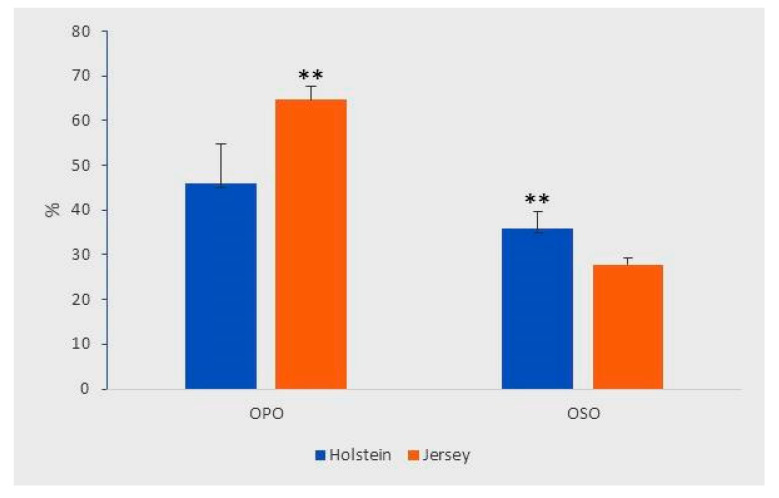
Comparison of OPO and OSO % in Holstein (*n* = 20) and Jersey milk (*n* = 10) samples. Error bars are SD; ** denotes highly significant difference between breeds (*p* < 0.01, *t*-test).

The % of OPO and OSO regioisomer was compared between Holstein and Jersey milk. It is of interest to note that Jersey milk contains a higher % of OPO regioisomer but a lower % of OSO regioisomer as compared to the Holstein milk (Figure 5). Besides, Figure 5 shows that inter-cow variation (as judged by the error bars) is much greater for Holstein than for Jersey breed, especially for the % of OPO. To our knowledge, this is the first report on the inter-breed difference in regioisomer proportion of milk TAGs; the underlying mechanisms remain to be investigated.

### 2.5. Inter-Cow Variation of Regioisomer Ratios

**Figure 6:**
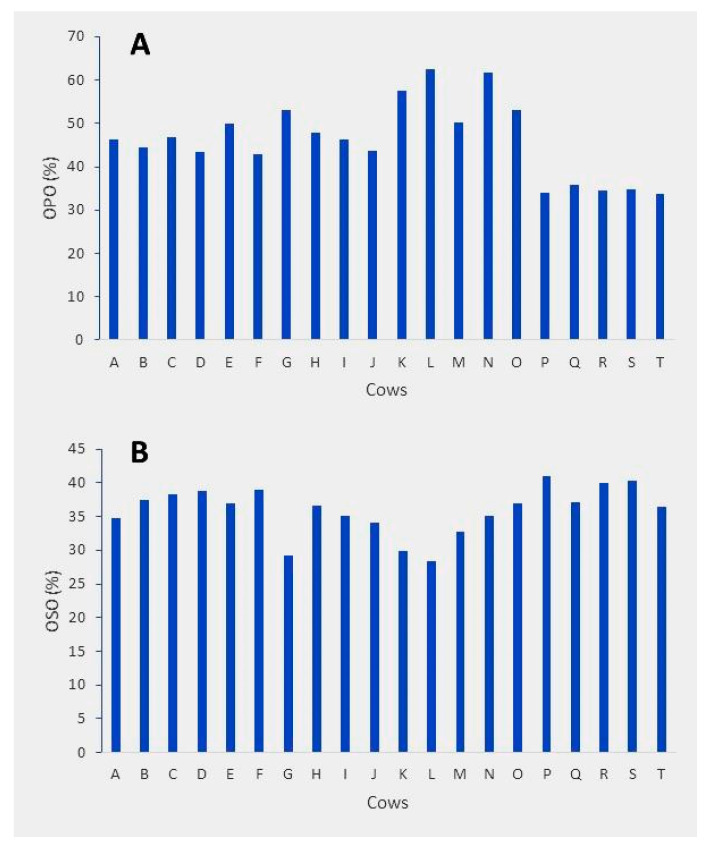
Variation of regioisomer OPO % (**A**) and OSO % (**B**) across the 20 milk samples of Holstein cows.

Detailed data on the % of OPO and OSO with the 20 individual Holstein cows are shown in Figure 6. The % of OPO ranges from 33 to 63, whereas that of OSO varies between 28 and 41, implying a greater inter-cow variation for OPO %. These results appear to suggest that the OPO % in bovine milk may be related to animal genotypes, since all the animals were from the same herd with the same feeding regime. Clearly, the rather small number of animal samples analyzed in this work does not allow us to estimate the heritability of this trait. Furthermore, the lower overall % of OSO as compared to OPO found in these samples is puzzling, and more work is needed to deepen our understanding on the regulation of milk lipid biosynthesis.

## 3. Materials and Methods

### 3.1. Milk Samples 

For method testing, one bulked bovine milk sample obtained from a local market and one human milk sample collected in Australia (with the consent of the donor) were used. For a preliminary inter-breed and inter-cow variation study, 20 and 10 milk samples from individual Holstein and Jersey cows (collected in 2015) were analyzed; the details of sample collection were described in our previous report [29]. All experimental cows were maintained in the research herd at the Department of Jobs, Precincts and Regions’ Ellinbank Center in Victoria, Australia, and the experiment was conducted in accordance with the Australian Code of Practice for the Care and Use of Animals for Scientific Purposes. Milk samples were transported to the laboratory on ice and kept at −80 °C before analysis.

### 3.2. TAG Standards

Regiopure TAG standards 1,2-olein-3-palmitin (OOP), 1,3-olein-2-palmitin (OPO), 1,2-olein-3-stearin (OOS), and 1,3-olein-2-stearin (OSO) were purchased from Larodan (Solna, Sweden). Solvents used for milk lipid extraction and mobile phase preparation were of MS or HPLC grade. Methanol and isopropanol were from Fisher Scientific (Ottawa, Canada), chloroform from Sigma-Aldrich (St. Louis, MO, USA), and acetonitrile and butanol from Merck (Kenilworth, NJ, USA). Ammonium formate (used as a mobile phase additive) was of analytical grade (Sigma-Aldrich, St. Louis, MO, USA). 

### 3.3. Lipid Extraction

A one-phase extraction method reported previously was used for milk lipid extraction [30]. Briefly, to 50 µL of milk (neat or diluted), 0.9 mL of solvent mix (butanol/methanol/chloroform, 3/5/4, *v*/*v*/*v*) was added. After a thorough mixing by vortex (for 30 s) and centrifugation for 10 min (13,000 *g*), the supernatant containing all lipids was analyzed directly by LC-MS without further clean up.

### 3.4. LC-MS Analysis

An Agilent 1290 UPLC system coupled to an LTQ-Orbitrap MS (Thermo Scientific, Waltham, MA, USA) was used for TAG quantification. Chromatographic separation of TAGs was achieved using an Acclaim C30 column (250 × 3 mm, 3 µm, Thermo Scientific) maintained at 30 °C. The mobile phase was composed of acetonitrile (A) and acetonitrile/IPA (10/90, *v*/*v*) containing 10 mM ammonium formate (B). The flow rate was 0.45 mL/min with a gradient elution of 10 to 80% B over 25, and then to 90% B from 25 to 30 min; the total run was 36 min. The injection volume was 5 µL.

An LTQ-Orbitrap mass spectrometer (Thermo Scientific) equipped with a heated electrospray ionization (HESI) source was used for TAG molecule detection. The heated capillary was maintained at 300 °C with a source heater temperature of 300 °C, and the sheath, auxiliary and sweep gases were, respectively, at 30, 15 and 5 units. The instrument was operated in positive ion mode (4.2 kV) with a full scan (120–1200 *m*/*z*) at a resolution of 60,000 (FT mode), followed by MS2 scans (in collision-induced dissociation mode) with collision energy of 35 eV. The precursor isolation width was set to 2 Da and a dynamic exclusion of 3 s was enabled.

### 3.5. Statistical Analysis

To compare the percentage of OPO and OSO between Holstein and Jersey cows, the mean values and the standard deviation (SD) for each cohort were presented. The data were also subjected to Student’s *t*-test (Microsoft Excel, Version 2008) for statistical comparison.

## 4. Conclusions

Using fragment ion ratios to determine the proportion of co-eluting regioisomers of TAGs proved to be feasible for milk samples. However, chromatographic separation of interfering isobaric species with C30 stationary phase is essential to ensure the accuracy of this approach. Application of this method to a small number of animals revealed that the proportion of regioisomer OPO and OSO differs between Holstein and Jersey milk; a large variation in OPO % was also observed across individual Holstein cows, suggesting a plausible genetic control of this trait.

## Figures and Tables

**Table 1:** Method repeatability (*n* = 3).

Sample Types	OPO (%)			OSO (%)		
	Mean	SD	RSD	Mean	SD	RSD
BM	53.7	0.8	1.5	29.4	0.7	2.4
BM (10× diluted)	52.1	0.6	1.2	27.9	0.5	1.8
HM	87.6	0.9	1.0	40.3	0.6	1.5
HM (10× diluted)	86.8	1.5	1.7	38.9	1.1	2.8

BM: bovine milk; HM: human milk.

## Supplementary Materials

The following are available online, Figure S1: Total ion chromatogram (TIC) of bovine milk (A) and human milk (B) lipids. Figure S2: Extracted ion chromatogram (EIC) of TAG 52:2 isomers of bovine milk after a gradient elution of 50 min.

## Sample Availability

Samples of the compounds are available from the authors.
