# Supplementary material for: Bovine Milk Triacylglycerol Regioisomer Ratio Shows Remarkable Inter-Breed and Inter-Cow Variation
Source: Molecules. 2021 Jun 28;26(13):3938. doi: 10.3390/molecules26133938 (PMC8271425; doi:10.3390/molecules26133938)
Supplement: Supplementary file 1 [file molecules-26-03938-s001.zip › molecules-1268171-supplementary.pdf]

Bovine Milk Triacylglycerol Regioisomer Ratio Shows Remarkable Inter-Breed and Inter-Cow Variation

Zhiqian Liu [1,*] and Simone Rochfort [1,2]

[1] Agriculture Victoria Research, AgriBio, 5 Ring Road, Bundoora, Victoria 3083, Australia;
Simone.Rochfort@agriculture.vic.gov.au

[2] School of Applied Systems Biology, La Trobe University, Bundoora, Victoria 3083, Australia

* Correspondence: Zhiqian.liu@agriculture.vic.gov.au; Tel: +61-3-9032-7134

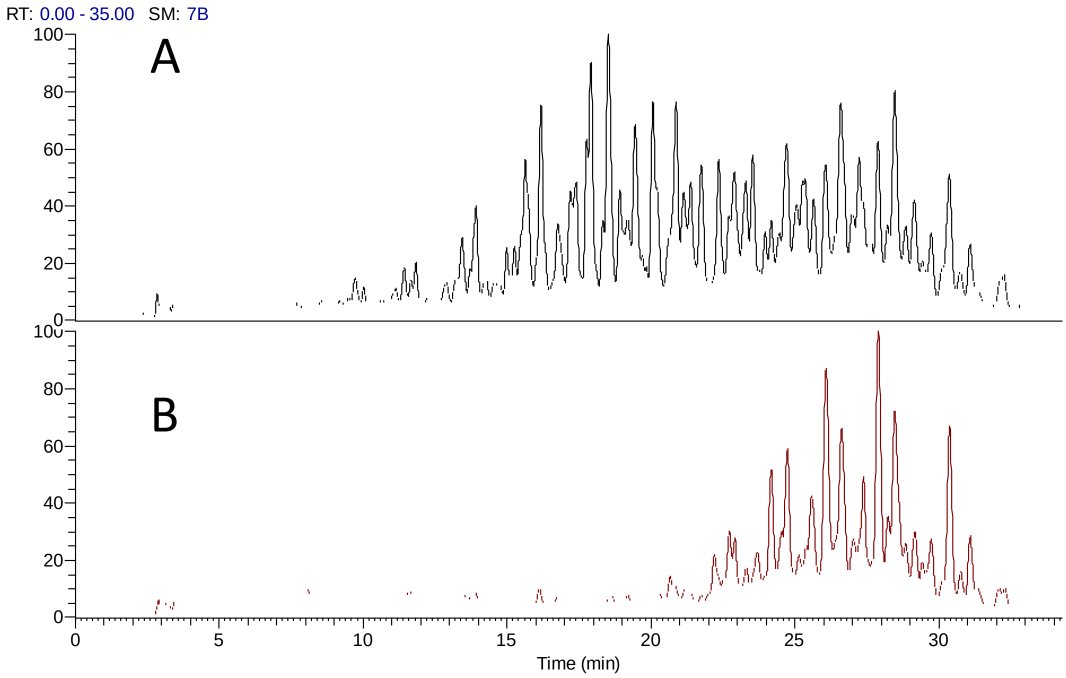

Figure S1. Total ion chromatogram (TIC) of bovine milk (A) and human milk (B) lipids.

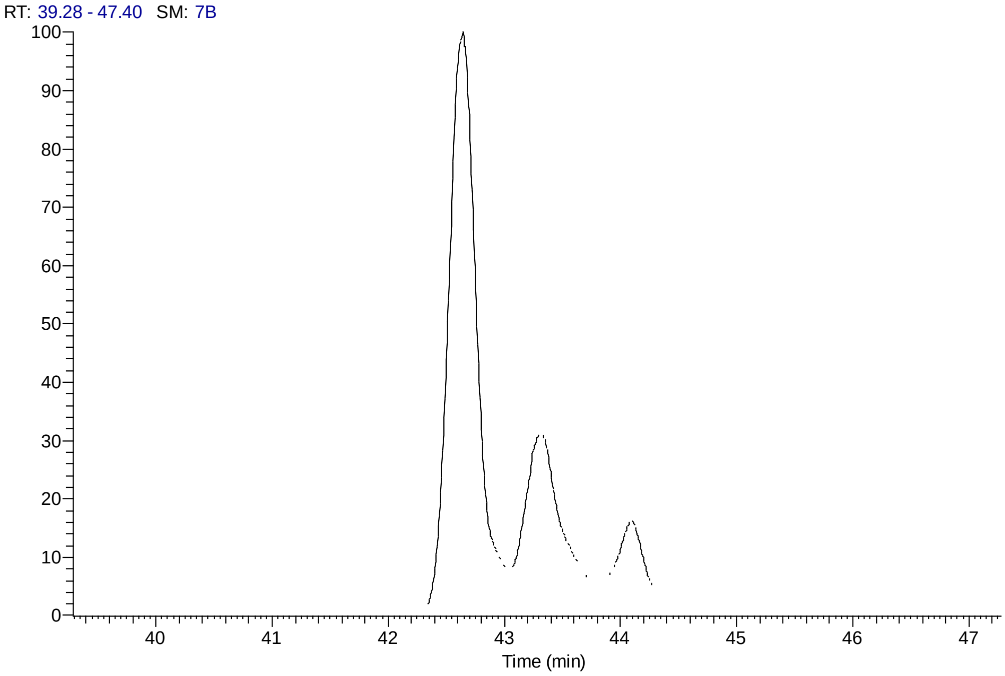

Figure S2. Extracted ion chromatogram (EIC) of TAG 52:2 isomers of bovine milk after a gradient elution of 50 min.
